# Differential Invariants of Measurements, and Their Relation to Central Moments

**DOI:** 10.3390/e22101118

**Published:** 2020-10-03

**Authors:** Eivind Schneider

**Affiliations:** Faculty of Science, University of Hradec Králové, Rokitanského 62, 50003 Hradec Králové, Czech Republic; eivind.schneider@uhk.cz

**Keywords:** measurement, information gain, thermodynamics, symplectic geometry, contact geometry, central moments, differential invariants, heat capacity

## Abstract

Due to the principle of minimal information gain, the measurement of points in an affine space *V* determines a Legendrian submanifold of $V\times V^{*}\times R$. Such Legendrian submanifolds are equipped with additional geometric structures that come from the central moments of the underlying probability distributions and are invariant under the action of the group of affine transformations on *V*. We investigate the action of this group of affine transformations on Legendrian submanifolds of $V\times V^{*}\times R$ by giving a detailed overview of the structure of the algebra of scalar differential invariants, and we show how the scalar differential invariants can be constructed from the central moments. In the end, we view the results in the context of equilibrium thermodynamics of gases, and notice that the heat capacity is one of the differential invariants.

## 1. Introduction

It has been known since the work of Gibbs [1,2] that thermodynamics can be formulated in the language of contact geometry. Because the fundamental thermodynamic relation takes the form of a contact structure on an odd-dimensional manifold, closed systems in thermal equilibrium correspond to Legendrian submanifolds with respect to this contact structure. More recently, some effort has been devoted towards studying an additional metric structure in the context of thermodynamics (see for example [3,4] and references therein).

Both the contact structure and the metric can be given statistical interpretations [5,6]. The principle of minimal information gain (similar to Jaynes’ principle of maximal entropy, [7]), applied to a random vector taking values in an affine space *V*, gives a contact structure on $V\times V^{*}\times R$. The variance of the random vector determines a metric on Legendrian submanifolds. In fact, for each integer k≥2, the *k*th central moment gives a symmetric *k*-form on Legendrian submanifolds [6].

In this framework, the group of affine transformations on *V* plays an important role, as it acts on $V\times V^{*}\times R$, and on the space of Legendrian submanifolds, while preserving the geometric structures mentioned. If we treat Legendrian submanifolds that are related by a transformation in this group as equivalent, it is clear that the important quantities are those that are invariant under this group action. Such quantities will be the main focus of this paper. In particular, we give a detailed description of the differential algebra of scalar differential invariants, building upon the work done in [8].

The paper is organized as follows. We start in Section 2 by recalling how the process of measuring points in an affine space leads to symplectic and contact geometry, following [6]. We explain how Legendrian submanifolds come equipped with symmetric *k*-forms corresponding to *k*th central moments. We end the section by discussing the action of the Lie group Aff(V) of affine transformations on *V*. In Section 3, we recall notions from the geometric theory of PDEs and explain the concept of scalar differential invariants. We show how the group Aff(V) acts on the jet spaces, and that its Lie algebra of vector fields is the largest Lie algebra of vector fields on *V* that preserves the central moments. In Section 4, we use the central moments to find generators for the field of rational scalar differential invariants. We compute the Hilbert and Poincaré functions for the field. We also find invariant derivations, and give a finite set of generators for the set of differential invariants, as a differential algebra. We compute differential syzygies for the case dimV=2. In Section 5, we apply our results to the thermodynamics of gases in equilibrium. We find a generating set of differential invariants and invariant derivations with respect to Aff(V), and we notice that the heat capacity (at constant pressure) is one of the fundamental scalar differential invariants. We also compute differential invariants with respect to two different subgroups of Aff(V) before we finish with a discussion of the significance of differential invariants.

## 2. From Random Vectors to Differential Geometry

We start by describing how the principle of minimal information gain applied to the measurement of points in an affine space leads to contact and symplectic geometry. We follow closely the approach of [6].

### 2.1. Measuring Points in Affine Spaces

A random vector is a map from a probability space to the affine space $V≃R^{n}$:

$$X:(\Omega ,A,\mu_{0})\to V$$

Here Ω is the sample space, A the σ-algebra of “events”, and $\mu_{0}$ a probability measure. We interpret *X* as a measurement of $x_{0}\in V$ if

$$E_{\mu_{0}}\left(X\right)=\int_{\Omega }Xd\mu_{0}=x_{0}.$$

By choosing an affine frame in *V*, the integral above can be defined coordinate-wise.

The measurement of a different vector x∈V is made by changing the probability measure from $\mu_{0}$ to μ, using the probability measure as a control parameter, in a way so that $E_{\mu }\left(X\right)=x$. We now find conditions that determine μ.

Assume first that $\mu_{0}$ is absolutely continuous with respect to μ. Then the Radon-Nikodym theorem implies that $d\mu =\rho d\mu_{0}$. We require ρ to satisfy the conditions

(1) $$E_{\mu }\left(X\right)=\int_{\Omega }X\rho d\mu_{0}=x,\;\int_{\Omega }\rho d\mu_{0}=1.$$

In addition we require μ to be the probability distribution “closest” to $\mu_{0}$ in the sense that it minimizes the information gain, or the Kullback-Leibler divergence:

$$I\left(\mu ,\mu_{0}\right)=\int_{\Omega }\rho \ln\rho \;d\mu_{0}$$

This is the principle of minimal information gain. It is similar to Jaynes’ maximum-entropy principle [7]. As Jaynes put it, it is the only unbiased assignment we can make. Note that Jensen’s inequality gives $I(\mu ,\mu_{0})\geq 0$. Moreover, we have $I(\mu ,\mu_{0})=0$ if ρ=1.

Minimizing $I(\mu ,\mu_{0})$ under the constraints (1) gives

(2) $$\rho =\frac{1}{Z(\lambda )}e^{\langle \lambda ,X\rangle }$$

where $\lambda \in V^{*}$ is a covector of Lagrange multipliers corresponding to the constraints given by the first equation of (1), and $Z\left(\lambda \right)=\int_{\Omega }e^{\langle \lambda ,X\rangle }d\mu_{0}$ is the partition function (see [6] for details). Thus ρ is determined by λ, and λ=0 implies ρ=1.

Let $D\subset V^{*}$ be a simply connected domain, containing 0, on which Z(λ) is defined and smooth. Due to the first condition of (1), we have $d_{\lambda }Z=Z\left(\lambda \right)x$. The differential $d_{\lambda }Z$ is an element in $T_{\lambda }^{*}D$ which can be identified with $V={\left(V^{*}\right)}^{*}$. In this identification, $x_{0}\in V$ is identified with $0\in T_{\lambda }^{*}D$. By defining H(λ)=−lnZ(λ), we get

(3) $$x=-d_{\lambda }H.$$

This defines an *n*-dimensional manifold

$$L=\left\{x=-d_{\lambda }H|\lambda \in D\right\}\subset V\times V^{*}.$$

By choosing an affine frame on *V* with origin $x_{0}$ (and its dual frame on $V^{*}$), we get coordinates $x^{i}$ on *V* and $\lambda_{i}$ on $V^{*}$. In these coordinates, the linear basis of the vector space associated to *V* can be identified with $d\lambda_{i}\in T_{\lambda }^{*}D$, and *L* is then given by the *n* equations $x^{i}=-H_{\lambda_{i}}$.

The space $V\times V^{*}$ is a symplectic space equipped with the symplectic form

(4) $$\omega =d\lambda_{i}\wedge dx^{i},$$

and *L* is a Lagrangian submanifold: ${\omega |}_{L}=0$. We use the Einstein summation convention, and sum over repeated indices.

Since the information gain $I(\mu ,\mu_{0})$ depends on ρ, and therefore on λ, it can be considered as a function on *L*. By using (2) we get

$$I\left(\mu ,\mu_{0}\right)=H\left(\lambda \right)+\langle \lambda ,x\rangle =H\left(\lambda \right)-\langle \lambda ,d_{\lambda }H\rangle$$

on *L*. If the point x∈V can be “measured”, then Equation (3) can be solved for λ. If λ(x) is a (local) solution, we may write

I(x)=H(λ(x))+〈λ(x),x〉.

Let *u* be a coordinate on R. We have the relation $I_{x^{i}}=\lambda_{i}$, so the submanifold

$$\tilde{L}=\left\{u=I\left(x\right),\lambda_{i}=I_{x^{i}}\left(x\right)\right\}\subset V\times V^{*}\times R$$

is Legendrian with respect to the contact form $\theta =du-\lambda_{i}dx^{i}$ on $V\times V^{*}\times R$.

The Legendrian submanifold $\tilde{L}$ depends on the initial random vector. In this way, the statistical object we started with is translated into a geometric object $\tilde{L}$. This shows that our model of physical measurements, based on random vectors and the principle of minimal information gain, is always accompanied by a form of thermodynamics, where the contact form $du-\lambda_{i}dx^{i}$ plays the role of the fundamental thermodynamic relation.

We note that in some cases, it is convenient to hide the information gain from our consideration and consider the Lagrangian submanifold $L\subset V\times V^{*}$ instead of $\tilde{L}\subset V\times V^{*}\times R$. We will switch between these two viewpoints.

### 2.2. Central Moments

Let $\tilde{L}$ continue to denote the Legendrian manifold corresponding to the random vector *X*. It can be parametrized either by the measured quantity x∈V or by the parameter $\lambda \in V^{*}$ (we will mostly use the latter choice).

The *k*th moment of *X* (with respect to μ) is defined by

$$m_{k}=\int_{\Omega }X^{⊗k}\rho d\mu_{0}.$$

It depends on λ and defines a symmetric tensor on $\tilde{L}$. We continue to use coordinates $x^{i}$ on *V* and $\lambda_{i}$ on $V^{*}$, coming from the choice of affine frame on *V* with origin $x_{0}$ and linear basis $d\lambda_{i}$. If we write $X=X^{i}d\lambda_{i}$, we get

$$\begin{array}{cc} m_{k} & =\left(\int_{\Omega }X^{i_{1}}\cdots X^{i_{k}}\rho d\mu_{0}\right)d\lambda_{i_{1}}⊗\cdots ⊗d\lambda_{i_{k}}=\frac{Z_{\lambda_{i_{1}}\cdots \lambda_{i_{k}}}}{Z}d\lambda_{i_{1}}⊗\cdots ⊗d\lambda_{i_{k}}. \end{array}$$

Assuming that the function *Z* is smooth, the differentiation is symmetric, and thus $m_{k}$ is a symmetric *k*-form defined on $\tilde{L}$. The equality

$$Z\int_{\Omega }X^{i_{1}}\cdots X^{i_{k}}\rho d\mu_{0}=Z_{\lambda_{i_{1}}\cdots \lambda_{i_{k}}}$$

can be shown by induction, using (2).

The *k*th central moment $\sigma_{k}$ of *X* is defined as the *k*th moment of $X-m_{1}$, where

$$m_{1}=\frac{Z_{\lambda_{i}}}{Z}d\lambda_{i}=-H_{\lambda_{i}}d\lambda_{i}=x^{i}d\lambda_{i}.$$

They are related through the formula

σk=∑i=0k(−1)k−ikimi·m1(k−i),

where the product is the symmetric tensor product. Note that the tensor $\sigma_{k}$ is completely determined by $\tilde{L}$, or even by the corresponding Lagrangian manifold $L\subset V\times V^{*}$.

The second central moment, or variance, determines a positive definite metric

$$\sigma_{2}=-H_{\lambda_{i}\lambda_{j}}d\lambda_{i}⊗d\lambda_{j}$$

on Legendrian submanifolds. It can also be given locally as $I_{x^{i}x^{j}}dx^{i}⊗dx^{j}$. Or if we consider only the Lagrangian submanifold $L\subset V\times V^{*}$, defined by *n* functions $x^{i}\left(\lambda \right)$, it can be given by $x_{\lambda_{j}}^{i}d\lambda_{i}⊗d\lambda_{j}$. Notice that the positive definiteness of $\sigma_{2}$ puts conditions on $\tilde{L}$. Thus, the measurement process does not lead to arbitrary Legendrian manifolds, but only to ones where the symmetric *k*-forms can be properly interpreted as central moments.

The metric $\sigma_{2}$ has been studied previously in different contexts. In particular it was treated by Ruppeiner [3,4] as a metric on *V* (in our notation). Ruppeiner was also interested in computing and giving a physical interpretation to its scalar curvature. However, as was pointed out in [6], and which we will discuss further in this paper, the group Aff(V) which appears naturally in this context is smaller than the full Lie pseudogroup of diffeomorphisms. As a result, the scalar curvature is not the most fundamental invariant. In [5], $\sigma_{2}$ was treated as a metric on Legendrian submanifolds of $V\times V^{*}\times R$ in the same way as we do in this paper. There, they also showed that it can be extended to a nondegenerate metric on $V\times V^{*}\times R$.

### 2.3. The Action of the Group of Affine Transformations

In the framework outlined above, we get quite naturally an action of the affine group Aff(V). One can view it as coming from the arbitrary choice of affine frame on *V*. The action extends to $V\times V^{*}\times R$ in the obvious way:

$$\left(x^{i},\lambda_{j},u\right)\mapsto \left(A_{k}^{i}x^{k}+B^{i},{\left(A^{-1}\right)}_{j}^{l}\lambda_{l},u\right),\;A\in GL\left(n\right),\;B\in R^{n}.$$

This Lie group action preserves the contact structure on $V\times V^{*}\times R$ (or the symplectic structure on $V\times V^{*}$). Thus, it also gives an action on the space of Legendrian (or Lagrangian) submanifolds. Moreover, the tensor $\sigma_{k}$ is preserved by Aff(V) for every k≥2. This will be discussed further in the next section. In particular, we will show in Theorem 2 in Section 3.2 that the Lie algebra of the Aff(V)-action is the largest Lie algebra of vector fields on *V* that preserves the tensor $\sigma_{2}$. Because of this, Aff(V) plays an important role in the theory of measurements, and this Lie group will be the focus of our attention in most of this paper.

We also have the invariant 0-form $\alpha_{0}=u$ and 1-form $du-\lambda_{i}dx^{i}$. The latter form vanishes when restricted to $\tilde{L}$, so we consider instead $\alpha_{1}=\lambda_{i}dx^{i}$ which also is invariant since *u*, and therefore du, is. Since $x^{i}=-H_{\lambda_{i}}$ on $\tilde{L}$, we have $dx^{i}=x_{\lambda_{j}}^{i}d\lambda_{j}=-H_{\lambda_{i}\lambda_{j}}d\lambda_{j}$. And thus

$$\alpha_{1}=x_{\lambda_{j}}^{i}\lambda_{i}d\lambda_{j}=-H_{\lambda_{i}\lambda_{j}}\lambda_{i}d\lambda_{j}.$$

**Remark** **1.** *Notice that there may be situations for which it is desirable to consider proper subgroups of* Aff(V) *only, perhaps because the space V has additional structure. For example, in the case where V is a vector space, it may be more appropriate to consider only the action of* GL(V) *. In this case, the regular kth moment* $m_{k}$ *will be invariant.*

## 3. Jets and PDEs

The theory of jet spaces lets us treat functions, sections of bundles, submanifolds of a fixed dimension and, more generally, solutions of PDEs geometrically. In particular it gives a transparent picture of the algebra of scalar differential invariants. We will use most of this section to fix notation and definitions, sufficient for our use, and refer to the standard literature (for example [9,10,11]) for details. We recommend [12] for a comprehensive introduction the theory of jet spaces and differential invariants, and [13] for a more concise overview. The paper [14] can be added to either one of these as an updated treatment of the theory of scalar differential invariants.

### 3.1. Jets

We have seen that the Legendrian submanifolds in $V\times V^{*}\times R$ can be represented, locally, by a function I(x) on *V*. Let $J^{k}\left(V\right)$ denote the space of *k*-jets of functions on *V*. It is a bundle over *V*. As coordinates on $J^{k}\left(V\right)$ we will use

$$x^{i},\;u,\;u_{x^{i}},\;u_{x^{i}x^{j}},\;...,\;u_{x^{i_{1}}\cdots x^{i_{k}}},\;i_{1}\leq \cdots \leq i_{k}.$$

We have

dimJk(V)=n+n+kk.

By identifying $u_{x^{i}}$ with $\lambda_{i}$ we get an identification of $J^{1}\left(V\right)$ with $V\times V^{*}\times R$, and the contact form on $V\times V^{*}\times R$ is identified with the contact form on $J^{1}\left(V\right)$. A Legendrian submanifold $\tilde{L}$ of $J^{1}\left(V\right)≃V\times V^{*}\times R$ can be prolonged canonically to an *n*-dimensional submanifold ${\tilde{L}}^{k}\subset J^{k}\left(V\right)$, by requiring that ${\tilde{L}}^{k}$ is an integral manifold of the Cartan distribution on $J^{k}\left(V\right)$.

Alternatively, we may remove information gain from the picture, and consider Lagrangian submanifolds of $V\times V^{*}$ with the symplectic form ω. (The information gain may be recovered later, up to an additive constant, by solving the system $I_{x^{i}}=\lambda_{i}$.)

Let $J^{k}\left(V\times V^{*},n\right)$ denote the space of *k*-jets of *n*-dimensional submanifolds of $V\times V^{*}$. Its dimension is given by

dimJk(V×V*,n)=n+nn+kn.

The symplectic form ω defines a PDE $E_{1}\subset J^{1}\left(V\times V^{*},n\right)$. A submanifold $L\subset V\times V^{*}$ is a Lagrangian submanifold if and only if its 1-jets are contained in $E_{1}$. Using coordinates $x^{i},\lambda_{j}$ on $V\times V^{*}$, a Lagrangian submanifold *L* is locally determined by *n* functions $x^{i}\left(\lambda \right)$ for i=1,...,n. (For thermodynamics of gases this corresponds to writing the internal energy and volume as functions of temperature and pressure.) Since $dx^{i}=x_{\lambda_{j}}^{i}d\lambda_{j}$ on *L*, the restriction of the symplectic form $\omega =d\lambda_{i}\wedge dx^{i}$ to *L* is given by

$${\omega |}_{L}=\sum_{i,j=1}^{n}x_{\lambda_{j}}^{i}d\lambda_{i}\wedge d\lambda_{j}.$$

Thus, the manifold *L* is Lagrangian (${\omega |}_{L}=0$) if and only if

(5) $$F_{ij}=x_{\lambda_{j}}^{i}-x_{\lambda_{i}}^{j}=0.$$

If we use the coordinates

$$\lambda_{i},\;x^{j},\;x_{\lambda_{i}}^{j},\;...,\;x_{\lambda_{i_{1}}\cdots \lambda_{i_{k}}}^{j},\;i_{1}\leq \cdots \leq i_{k},$$

on $J^{k}\left(V\times V^{*},n\right)$, it is clear that the Equation (5) define $E_{1}\subset J^{1}\left(V\times V^{*},n\right)$.

By differentiating the n2 equations $x_{\lambda_{j}}^{i}=x_{\lambda_{i}}^{j}$ with respect to the variables $\lambda_{1},...,\lambda_{n}$ we get nn2 additional equations of order two. We add these to the original set of first-order equations, and we denote the corresponding manifold in $J^{2}\left(V\times V^{*},n\right)$ by $E_{2}$. Similarly, we get submanifolds $E_{k}\subset J^{k}\left(V\times V^{*},n\right)$ for every positive integer *k* by adding all derivatives (of appropriate order) of the first-order equations. We will also use the notation $E_{0}=J^{0}\left(V\times V^{*},n\right)=V\times V^{*}$.

By counting the number of equations, we easily get the following statement.

**Theorem** **1.** *The dimension of $E_{k}$ is given by*

dimEk=dimJk+1(V)−1=n+n+k+1n−1,

*for k≥0.*

Note that by throwing away the information gain from the picture, we get a natural projection $J^{k+1}\left(V\right)\to E_{k}\subset J^{k}\left(V\times V^{*},n\right)$, which is reflected in the counting above.

The central moment $\sigma_{k}$ can be interpreted as a horizontal symmetric form on $E_{k}$ (or on $J^{k}\left(V\right)$). Loosely speaking, this means that it is defined by the same formula as before, only that its coefficients is now considered as functions on $E_{k}$ (or on $J^{k}\left(V\right)$). For example, in the formula $x_{\lambda_{j}}^{i}d\lambda_{i}⊗d\lambda_{j}$ for $\sigma_{2}$, the coefficient $x_{\lambda_{j}}^{i}$ is viewed as a function on $E_{k}$ rather than a function on $V^{*}$. Indeed, it is one of the coordinate functions on $J^{k}\left(V\times V^{*},n\right)$. If a Lagrangian manifold is given, $\sigma_{k}$ can be restricted to the *k*th prolongation of *L* and get back its original meaning. In this way, the theory of jet spaces lets us work with functions and tensors on *L* that depend on $x^{i}\left(\lambda \right)$ and their derivatives without specifying *L*.

### 3.2. The Action of the Affine Group on $J^{k}\left(V\right)$ and $E_{k}$

In Section 2.3 we explained how Aff(V) acts on $V\times V^{*}\times R$. The action on *V* induces uniquely an action on $V\times V^{*}\times R$ which preserves the contact form $\theta =du-\lambda_{i}dx^{i}$. This also gives a group action of Aff(V) on $J^{1}\left(V\right)$. More generally, a transformation on $J^{0}\left(V\right)$ uniquely induces a transformation on $J^{k}\left(V\right)$ for every positive integer *k* (it transforms functions on *V* and, as a consequence, their *k*-jets). In this way, the Aff(V)-action can be prolonged to $J^{k}\left(V\right)$. The same is true for the corresponding Lie algebra of vector fields defined on $J^{0}\left(V\right)$.

We illustrate this by prolonging a vector field on the base of $J^{0}\left(V\right)=V\times R$ to a vector field on $J^{2}\left(V\right)$, and refer to [10] for a more general treatment. Consider the vector field $X=a^{i}\left(x\right)\partial_{x^{i}}$ on V×R. The unique vector field on $J^{1}\left(V\right)$ that preserves the Cartan distribution and projects to *X* is given by

$$X^{\left(1\right)}=a^{i}\left(x\right)\partial_{x^{i}}-a_{x^{j}}^{s}\left(x\right)u_{x^{s}}\partial_{u_{x^{j}}}.$$

Note that when $a^{i}$ are affine functions, this corresponds exactly to the Aff(V)-action described in Section 2.3. The prolongation of *X* to $J^{2}\left(V\right)$ is given by

$$X^{\left(2\right)}=a^{i}\partial_{x^{i}}-a_{x^{j}}^{s}u_{x^{s}}\partial_{u_{x^{j}}}-\left(D_{x^{m}}\left(u_{x^{s}}\right)a_{x^{l}}^{s}+D_{x^{l}}\left(u_{x^{s}}\right)a_{x^{m}}^{s}+u_{s}a_{x^{l}x^{m}}^{s}\right)\partial_{u_{x^{l}x^{m}}}.$$

In this formula l≤m is assumed in the summation, and $D_{x^{i}}$ is the total derivative operator. The total derivative is used in order to avoid expressions such as $u_{x^{2}x^{1}}$, which is not a function on $J^{2}\left(V\right)$. In the case m≥s, we have $D_{x^{m}}\left(u_{x^{s}}\right)=u_{x^{s}x^{m}}$. For m≤s, we have $D_{x^{m}}\left(u_{x^{s}}\right)=u_{x^{m}x^{s}}$.

**Remark** **2.** In a similar way we get an Aff(V)-action on $J^{k}\left(V\times V^{*},n\right)$ for k≥0. Notice that since Aff(V) preserves the symplectic structure on $V\times V^{*}$, it preserves the subset $E_{k}\subset J^{k}\left(V\times V^{*},n\right)$, and we may consider the Aff(V)-action on $E_{k}$.

When we say that $\sigma_{k}$ is Aff(V)-invariant, we mean that it is invariant as a horizontal symmetric form with respect to the Aff(V)-action on $J^{k}\left(V\right)$. More precisely, we have $\varphi^{*}\sigma_{k}=\sigma_{k}$ for every φ∈Aff(V), where φ is understood as a transformation $J^{k}\left(V\right)\to J^{k}\left(V\right)$.

Now, let us see which conditions we get on the functions $a^{i}$ if we require the vector field $X^{\left(2\right)}$ from above to preserve the variance $\sigma_{2}=D_{x^{i}x^{j}}\left(u\right)dx^{i}⊗dx^{j}$ (i.e. $L_{X^{\left(2\right)}}\sigma_{2}=0$). Here $D_{x^{i}x^{j}}=D_{x^{j}}\circ D_{x^{i}}$. We have

$$\begin{array}{c} L_{X^{\left(2\right)}}\left(\sigma_{2}\right)=X^{\left(2\right)}\left(D_{x^{i}x^{j}}\left(u\right)\right)dx^{i}⊗dx^{j}+D_{x^{i}x^{j}}\left(u\right)\left(L_{X^{\left(2\right)}}\left(dx^{i}\right)⊗dx^{j}+dx^{i}⊗L_{X^{\left(2\right)}}\left(dx^{j}\right)\right), \end{array}$$

and

$$\begin{array}{cc} X^{\left(2\right)}\left(u_{x^{i}x^{j}}\right) & =-(D_{x^{j}}\left(u_{x^{s}}\right)a_{x^{i}}^{s}+D_{x^{i}}\left(u_{x^{s}}\right)a_{x^{j}}^{s}+u_{s}a_{x^{i}x^{j}}^{s}),\\ L_{X^{\left(2\right)}}\left(dx^{s}\right) & =d\left(i_{X_{f}^{\left(2\right)}}dx^{s}\right)=d\left(a^{s}\right)=a_{x^{i}}^{s}dx^{i}. \end{array}$$

The terms of $L_{X^{\left(2\right)}}\left(\sigma_{2}\right)$ that depend on first-order derivatives of $a^{i}$ cancel, and we see that $L_{X^{\left(2\right)}}\left(\sigma_{2}\right)$ vanishes if and only if all second-order partial derivatives of $a^{i}$ vanish, implying that $a^{i}$ are affine functions on *V*. We get the following statement.

**Theorem** **2.** *Let $X=a^{i}\left(x\right)\partial_{x^{i}}$ be a vector field on V×R. Then X preserves $\sigma_{2}$ (i.e. $L_{X^{\left(2\right)}}\left(\sigma_{2}\right)=0$) if and only if X is an affine vector field.*

The fact that affine vector fields preserve the tensors $\sigma_{k}$ and that, due to the above theorem, they are the only vector fields on *V* to do so, emphasizes the importance of the Aff(V)-action in the context of measurements.

### 3.3. Differential Invariants

Since Aff(V) acts on $J^{k}\left(V\right)$, we can look for functions on $J^{k}\left(V\right)$ that are Aff(V)-invariant.

**Definition** **1.** *A (scalar) differential invariant of order k is a function on $J^{k}\left(V\right)$ which is constant on Aff(V)-orbits.*

Notice that a differential invariant of order *k* is also a differential invariant of order k+1. This determines a filtration on the algebra of differential invariants.

Due to the natural projection $J^{k+1}\left(V\right)\to E_{k}\subset J^{k}\left(V\times V^{*},n\right)$, any function on $E_{k}$ gives a function on $J^{k+1}\left(V\right)$. Essentially, the only thing we loose by considering functions on $E_{k}$, is the zero-order invariant *u* corresponding to the information gain. In the remaining sections we will mostly consider Lagrangian submanifolds of $V\times V^{*}$, and therefore we will usually not mention the information gain explicitly in our description of differential invariants. Notice also that, by our definition, an invariant defined on $E_{k}$ will be of order k+1 and not, in general, of order *k*.

We follow [14] and consider the field of rational differential invariants. This is sufficient for separating orbits in general position as the group action, and the PDE on which it acts, is algebraic. More precisely, for every k≥1, there is a Zariski-open subset of $E_{k-1}$ on which the field of rational differential invariants of order *k* separate orbits. The set of rational differential invariants can also be considered as a differential algebra. By using invariant derivations, the differential algebra of differential invariants can be finitely generated.

## 4. Scalar Differential Invariants

In this section, we construct scalar differential invariants from the symmetric tensors $\sigma_{k}$ and the 1-form $\alpha_{1}$ which was discussed in Section 2.3. We end up with a transcendence basis for the field of differential invariants of order *k*, for every *k*, and we find a finite set of generators for the differential algebra of differential invariants. Differential syzygies are computed for the case dimV=2.

As is common when looking for differential invariants, we will be satisfied with obtaining a number of independent differential invariants of order *k* equal to the codimension of an orbit in general position in $E_{k-1}$. In other words, we will generate a differential algebra of differential invariants which, for each order *k*, contains a transcendence basis for the field of differential invariants of order *k*. In general, the field of rational differential invariants of order *k* will be an algebraic extension of the field generated by the transcendence basis. In this section, we will focus on Lagrangian submanifolds of $V\times V^{*}$ and therefore, in most cases, disregard the differential invariant of order zero corresponding to the information gain.

**Remark** **3.** *All results in this section may be useful also if we want to consider a subgroup G⊂Aff(V), for example due to preservation of additional structure on V. All scalar and tensorial invariants will be invariant also with respect to G. The essential changes in the algebra of scalar differential invariants will occur on the level of third-order invariants, where new invariants will appear.*

### 4.1. From Invariant Symmetric Forms to Scalar Invariants

We will now find the scalar differential invariants. We use $\alpha_{1}$, $\sigma_{2}$ and $\sigma_{3}$ to construct an invariant frame on $L\subset V\times V^{*}$.

1. The symmetric 2-form $\sigma_{2}$ is nondegenerate, and is used to construct a vector field $v_{1}=\sigma_{2}^{-1}\left(\alpha_{1}\right)$.
2. By using $\sigma_{2}$ again, we turn the symmetric 2-form $i_{v_{1}}\sigma_{3}$ into a linear map A:TL→TL.
3. We use *A* to define the n−1 additional vector fields $v_{i}=A^{i-1}v_{1}$ for i=2,...,n.

We require that $v_{1},...,v_{n}$ constitute a frame on *L*. This puts conditions on *A*, and therefore on the 2-jets of *L*. These conditions hold on a Zariski-open subset of $E_{2}$.

Note that all the steps above can be taken without fixing the manifold *L* if we treat $\alpha_{1}$ and $\sigma_{k}$ for k≥2 as horizontal symmetric forms on $E_{k}$. The inverse of $\sigma_{2}=s^{ij}d\lambda_{i}⊗d\lambda_{j}$ is then given by $\sigma_{2}^{-1}=s_{ij}D_{\lambda_{i}}⊗D_{\lambda_{j}}$, where $D_{\lambda_{i}}$ is the total derivative. Note that $d\lambda_{i}\left(D_{\lambda_{j}}\right)=\delta_{i}^{j}$. Thus, $v_{1}=\lambda_{i}D_{\lambda_{i}}$ and $v_{i}=v_{ij}D_{\lambda_{j}}$, where $v_{ij}$ are (rational) functions on $E_{2}$ determined by the algorithm above. These are horizontal vector fields on $E_{\infty }$.

4. The functions $\sigma_{k}\left(v_{i_{1}},...,v_{i_{k}}\right)$ are rational, scalar differential invariants.

**Remark** **4.** *Note that this idea has previously been used, with slight modifications, when considering subgroups of Aff(V). Differential invariants of the actions of the Euclidean group and the general linear group on functions were considered in [15,16], respectively.*

The next step is justifying that we can find, among these differential invariants that we have constructed, a transcendence basis for the field of differential invariants of order *k*, for each order *k*.

**Lemma** **1.** *There is only one independent differential invariant of order 2. It can be given in coordinates by*

$$\sigma_{2}\left(v_{1},v_{1}\right)=x_{\lambda_{j}}^{i}\lambda_{i}\lambda_{j}.$$

**Proof.**  Consider the action of Aff(V) on the 2-jet of the information gain I(x) at the point $x=x_{0}$. By using translations on *V*, we may set $x_{0}=0$, so that the 2-jet is given by $j_{0}^{2}\left(I\right)\left(x\right)=I\left(0\right)+a_{i}x^{i}+a_{ij}x^{i}x^{j}$. The linear and quadratic terms can be encoded in terms of a one-form and a nondegenerate metric, respectively (in fact, by $\alpha_{1}$ and $\sigma_{2}$). The action by GL(V) (the stabilizer of 0) preserves the degree of monomials, and we can use it to normalize the quadratic terms to get $j_{0}^{2}\left(I\right)\left(x\right)=I\left(0\right)+b_{i}x^{i}+\sum_{i}{\left(x^{i}\right)}^{2}$. Next, we use the stabilizer O(V) of the quadratic form, and rotate the expression into $j_{0}^{2}\left(I\right)\left(x\right)=I\left(0\right)+cx^{1}+\sum_{i}{\left(x^{i}\right)}^{2}$. We have used up all the freedom the group gives us, and the 2-jet is brought to normal form. There is only one free constant *c*, in addition to I(0), so there is at most one invariant of second order in addition to the zero-order information gain, namely the length of the one-form (or its square). □

Before we consider the differential invariants of third order, we consider the invariants of higher order, which is an easier task. At points in general position in $E_{2}$, the horizontal vector fields $v_{1},...,v_{n}$ are linearly independent. Notice that the function $\sigma_{k}\left(D_{\lambda_{i_{1}}},...,D_{\lambda_{i_{k}}}\right)$ is a sum of $x_{\lambda_{i_{2}}\cdots \lambda_{i_{k}}}^{i_{1}}$ and lower-order terms. Since the coefficients of $v_{i}$ depend on $E_{2}$, the invariants $\sigma_{4}\left(v_{i_{1}},...,v_{i_{4}}\right)$, with $i_{1}\leq \cdots \leq i_{4}$, are algebraically independent when restricted to a generic fiber of $E_{3}\to E_{2}$. This argument is easily extended to k>4, and we obtain the following statement.

**Lemma** **2.** *The differential invariants $\sigma_{k}\left(v_{i_{1}},...,v_{i_{k}}\right)$, with $i_{1}\leq \cdots \leq i_{k}$ and k≥4, are algebraically independent. Moreover, for each k≥4, the number of independent functions among them, when restricted to a generic fiber of $E_{k-1}\to E_{2}$, is equal to the dimension of the fiber.*

We deduce from the lemma that the Aff(V)-action is locally free on $E_{2}$, meaning that the dimension of an orbit in general position in $E_{2}$ is equal to $\dim\mathrm{Aff}\left(V\right)=n^{2}+n$. Since we have dimE2=n+n+33−1, the codimension of an orbit in general position is given by

$$\dim E_{2}-\dim\mathrm{Aff}\left(V\right)=\frac{n^{3}+11n}{6}.$$

Therefore, in addition to the invariant of order two, there exist $\frac{n^{3}+11n-6}{6}$ algebraically independent differential invariants of order three. We will verify that we have found all of them.

It is clear from the construction of the invariant frame $\left\{v_{1},...,v_{n}\right\}$ that we have the relations

$$i_{v_{i}}\sigma_{2}=i_{v_{i-1}}i_{v_{1}}\sigma_{3},\;i=2,...,n.$$

Note how this is consistent with the fact that there is only one second-order invariant $\sigma_{2}\left(v_{1},v_{1}\right)$. It also follows that there will be relations between $\sigma_{3}\left(v_{1},v_{i},v_{j}\right)$:

(6) $$\sigma_{3}\left(v_{1},v_{i-1},v_{j}\right)=\sigma_{3}\left(v_{1},v_{i},v_{j-1}\right),\;i,j=2,..,n.$$

In two dimensions this holds trivially, since $\sigma_{3}$ is symmetric. In three dimensions the additional relation takes the form

$$\sigma_{3}\left(v_{1},v_{1},v_{3}\right)=\sigma_{3}\left(v_{1},v_{2},v_{2}\right).$$

In four dimensions we get two additional relations:

$$\sigma_{3}\left(v_{1},v_{1},v_{4}\right)=\sigma_{3}\left(v_{1},v_{2},v_{3}\right),\;\sigma_{3}\left(v_{1},v_{2},v_{4}\right)=\sigma_{3}\left(v_{1},v_{3},v_{3}\right)$$

For dimV=n≥3 we get n−12 relations between the n+23 components of $\sigma_{3}$. The difference is $\frac{n^{3}+11n-6}{6}$.

**Lemma** **3.** *The algebraic relations between the n+23 differential invariants $\sigma_{k}\left(v_{i},v_{j},v_{k}\right)$, with i≤j≤k, are exactly the n−12 relations given by (6).*

**Proof.**  It is sufficient to prove that there are n+23−n−12=n3+11n−66 algebraically independent functions in this set of differential invariants. We will do this by restricting to a suitable $\frac{n^{3}+11n-6}{6}$-dimensional submanifold in $E_{2}$, and show that there are $\frac{n^{3}+11n-6}{6}$ independent functions among the invariants, also after restricting to this submanifold. After the appropriate submanifold is found, the statement of the lemma follows quite easily.

First we restrict the invariants to the fiber of $E_{2}\to E_{1}$ given by

$$\lambda_{1}=1,\;\lambda_{2}=0,\;\cdots ,\;\lambda_{n}=0,\;x^{i}=0,\;x_{\lambda_{j}}^{i}=\delta^{ij},\;i,j=1,...,n.$$

On this fiber, the expressions for the tensors $\alpha_{1}$ and $\sigma_{2}$ are significantly simplified:

$$\alpha_{1}=d\lambda_{1},\;\sigma_{2}=\sum_{i=1}^{n}{\left(d\lambda_{i}\right)}^{2}.$$

We restrict further to a submanifold of this fiber, of codimension n−12, by setting

$$x_{\lambda_{i}\lambda_{j}}^{1}=0,\;j=2+i,...,n,\;i=1,...,n-2.$$

On this subset, we have

$$\begin{array}{c} v_{1}=D_{\lambda_{1}},\;v_{2}=x_{\lambda_{1}\lambda_{1}}^{1}D_{\lambda_{1}}+x_{\lambda_{1}\lambda_{2}}^{1}D_{\lambda_{2}},\\ v_{3}=\left({\left(x_{\lambda_{1}\lambda_{1}}^{1}\right)}^{2}+{\left(x_{\lambda_{1}\lambda_{2}}^{1}\right)}^{2}\right)D_{\lambda_{1}}+x_{\lambda_{1}\lambda_{2}}^{1}\left(x_{\lambda_{1}\lambda_{1}}^{1}+x_{\lambda_{2}\lambda_{2}}^{1}\right)D_{\lambda_{2}}+x_{\lambda_{1}\lambda_{2}}^{1}x_{\lambda_{2}\lambda_{3}}^{1}D_{\lambda_{3}},\;... \end{array}$$

and, in general, the only nonzero components of $v_{k}$ are in the $\lambda_{i}$-directions for i=1,...,k. Moreover, the coefficients for $v_{k}$ depend only on the variables $x_{\lambda_{k-1}\lambda_{k-1}}^{1}$ and $x_{\lambda_{k-1}\lambda_{k}}^{1}$ in addition to the variables of which $v_{k-1}$ depend. This allows for an induction argument, since the expression for $v_{i}$ is independent of n=dimV. We have

$$\sigma_{3}=D_{\lambda_{j}\lambda_{k}}\left(x^{i}\right)\;d\lambda_{i}⊗d\lambda_{j}⊗d\lambda_{k}.$$

For dimV=2 we get

$$\begin{array}{c} \sigma_{3}\left(v_{1},v_{1},v_{1}\right)=x_{\lambda_{1}\lambda_{1}}^{1},\;\sigma_{3}\left(v_{1},v_{1},v_{2}\right)={\left(x_{\lambda_{1}\lambda_{1}}^{1}\right)}^{2}+{\left(x_{\lambda_{1}\lambda_{2}}^{1}\right)}^{2},\\ \sigma_{3}\left(v_{1},v_{2},v_{2}\right)={\left(x_{\lambda_{1}\lambda_{1}}^{1}\right)}^{3}+2x_{\lambda_{1}\lambda_{1}}^{1}{\left(x_{\lambda_{1}\lambda_{2}}^{1}\right)}^{2}+{\left(x_{\lambda_{1}\lambda_{2}}^{1}\right)}^{2}x_{\lambda_{2}\lambda_{2}}^{1},\\ \sigma_{3}\left(v_{2},v_{2},v_{2}\right)={\left(x_{\lambda_{1}\lambda_{1}}^{1}\right)}^{4}+3{\left(x_{\lambda_{1}\lambda_{1}}^{1}\right)}^{2}{\left(x_{\lambda_{1}\lambda_{2}}^{1}\right)}^{2}+3x_{\lambda_{1}\lambda_{1}}^{1}{\left(x_{\lambda_{1}\lambda_{2}}^{1}\right)}^{2}x_{\lambda_{2}\lambda_{2}}^{1}+{\left(x_{\lambda_{1}\lambda_{2}}^{1}\right)}^{3}x_{\lambda_{2}\lambda_{2}}^{2}. \end{array}$$

These four functions are clearly independent, and thus the lemma is proved in the case dimV=2. With an appropriate choice of ordering of the functions $\sigma_{3}\left(v_{i},v_{j},v_{k}\right)$, each new function depends on the same variables as the previous function in addition to $x_{\lambda_{j}\lambda_{k}}^{i}$, for 1≤i≤j≤k≤2.

Due to the particular form of $\sigma_{3}$ and $v_{1},...,v_{n}$, when restricted to our submanifold, it is clear that this type of pattern will continue. By extending the sequence of functions, with one at a time, each variable $x_{\lambda_{j}\lambda_{k}}^{i}$ will be introduced with the function $\sigma_{3}\left(v_{i},v_{j},v_{k}\right)$, where j=i,...,k and i=1,...,k and k=1,...,n (this also indicates the appropriate ordering). There are two possible obstructions to this, and we have to justify that they are, in fact, not obstructions.

Firstly, the function $\sigma_{3}\left(v_{1},v_{1},v_{3}\right)$ will depend on the same variables as the four we found above, in addition to $x_{\lambda_{1}\lambda_{3}}^{1}$. This new variable is zero on our submanifold, and thus $\sigma_{3}\left(v_{1},v_{1},v_{3}\right)$ is not independent from the previous ones. However, this is exactly what we want, as we are already aware of the relation $\sigma_{3}\left(v_{1},v_{1},v_{3}\right)=\sigma_{3}\left(v_{1},v_{2},v_{2}\right)$. The situation is similar for $\sigma_{3}\left(v_{1},v_{1},v_{4}\right)$ and $\sigma_{3}\left(v_{1},v_{2},v_{4}\right)$, and so on.

Secondly, even though the derivations depend in nontrivial ways on the variables, this fact does not interfere with the pattern. The potential new variables coming from the derivations will always be introduced (to our sequence of functions) with $\sigma_{3}\left(v_{1},v_{i-1},v_{i}\right)$, and these new variables would be $x_{\lambda_{i-1}\lambda_{i-1}}^{1}$ and $x_{\lambda_{i-1}\lambda_{i}}^{1}$. The first one was introduced already by $\sigma_{3}\left(v_{1},v_{i-1},v_{i-1}\right)$, and the second is exactly the one that should be introduced here, as it is equal to $\sigma_{3}\left(D_{\lambda_{1}},D_{\lambda_{i-1}},D_{\lambda_{i}}\right)$. The fact that $x_{\lambda_{1}\lambda_{2}}^{1}$ is squared in $\sigma_{3}\left(v_{1},v_{1},v_{2}\right)$ is a consequence of this. □

These lemmas imply that we have in the following sense constructed a complete set of rational differential invariants.

**Theorem** **3.** *For every order k, the field generated by $\sigma_{2}\left(v_{1},v_{1}\right)$ and $\sigma_{j}\left(v_{i_{1}},...,v_{i_{j}}\right)$, where $i_{1}\leq \cdots \leq i_{j}$ and j=3,...,k generates a field of differential invariants with transcendence degree equal to the codimension of an Aff(V)-orbit in general position in $E_{k-1}$.*

Let $s_{k}$ denote the codimension of an Aff(V)-orbit in $J^{k}\left(V\right)$, in general position, for k≥0. This number is the same as the transcendence degree of the field of rational scalar differential invariants of order *k*. The Hilbert function for the filtered field of differential invariants is defined as $H_{k}=s_{k}-s_{k-1}$ for k≥1, and $H_{0}=s_{0}$. From the discussion above we deduce the following statement.

**Theorem** **4.** *The Hilbert function for the field of differential invariants is given by*

H0=1,H1=0,H2=1,H3=n3+11n−66,Hk=n+k−1k,k≥4.

Note that for n=2, the formula for $H_{3}$ coincides with that for $H_{k}$, as both gives 4.

We define the Poincaré function corresponding to the Hilbert function by the series $P\left(z\right)=\sum_{k=0}^{\infty }H_{k}z^{k}$ which converges to a rational function for |z|<1.

**Theorem** **5.** *The Poincaré function for the field of differential invariants is given by*

$$P\left(z\right)={\left(1-z\right)}^{-n}-\frac{z}{2}\left(\left(n-1\right)\left(n-2\right)z^{2}+\left(n+2\right)\left(n-1\right)z+2n\right).$$

### 4.2. The Differential Algebra of Differential Invariants

The differential algebra of differential invariants is finitely generated (see [14] and references therein). The invariant horizontal vector fields $v_{1},...,v_{n}$ act on the differential invariants as derivations, resulting in new differential invariants which, in general, are of higher order. It is clear that we get, by taking invariant derivatives of the *k*th-order invariants $\sigma_{k}\left(v_{i_{1}},...,v_{i_{k}}\right)$, a set of $H_{k+1}$ independent differential invariants of order k+1, for k≥4 (since the coefficients of the derivations $v_{i}$ are only of order 3). See for example [12] (Theorem 5.48). Thus, the algebra of differential invariants can be generated by the differential invariants of order 4, together with the invariant derivations.

**Theorem** **6.** *The differential algebra of scalar differential invariants is generated by the invariant derivations $v_{1},...,v_{n}$ and the scalar invariants $\sigma_{2}\left(v_{1},v_{1}\right)$, $\sigma_{3}\left(v_{i},v_{j},v_{k}\right)$, and $\sigma_{4}\left(v_{i},v_{j},v_{k},v_{l}\right)$.*

Note that this generating set of differential invariants is not minimal. As explained in in the previous section, some of the invariants $\sigma_{3}\left(v_{i},v_{j},v_{k}\right)$ are algebraically related to each other. Moreover, many of the invariants $\sigma_{4}\left(v_{i},v_{j},v_{k},v_{l}\right)$ can be constructed by computing invariant derivatives of the third-order invariants. In particular, when n=2 the algebra of differential invariants can be generated by $v_{1},v_{2}$ and the differential invariants of order three.

### 4.3. Differential Syzygies for dimV=2

The algebra of differential invariants is not a freely generated differential algebra; there are differential syzygies among the generators. We will find the syzygies in the simplest case, when dimV=2. This will also let us sharpen Theorem 6 for this particular case.

Let us use the notation

$$\begin{array}{c} I_{21}=\sigma_{2}\left(v_{1},v_{1}\right),\;I_{22}=\sigma_{2}\left(v_{1},v_{2}\right),\;I_{23}=\sigma_{2}\left(v_{2},v_{2}\right),\;I_{31}=\sigma_{3}\left(v_{1},v_{1},v_{1}\right),\\ I_{32}=\sigma_{3}\left(v_{1},v_{1},v_{2}\right),\;I_{33}=\sigma_{3}\left(v_{1},v_{2},v_{2}\right),\;I_{34}=\sigma_{3}\left(v_{2},v_{2},v_{2}\right). \end{array}$$

We have $I_{22}=I_{31}$ and $I_{23}=I_{32}$. In order to write the differential syzygies in a relatively compact form, it will be useful to have the following definitions:

$$\begin{array}{c} J_{1}=\frac{I_{21}I_{33}-I_{22}I_{32}}{I_{21}I_{23}-I_{22}^{2}},\;J_{2}=\frac{I_{22}I_{33}-I_{23}I_{32}}{I_{21}I_{23}-I_{22}^{2}},\;J_{3}=\frac{I_{21}I_{34}-I_{22}I_{33}}{I_{21}I_{23}-I_{22}^{2}},\;J_{4}=\frac{I_{22}I_{34}-I_{23}I_{33}}{I_{21}I_{23}-I_{22}^{2}} \end{array}$$

When dimV=2, the third-order invariants are sufficient to generate the whole algebra. Furthermore, if we compute invariant derivatives of $I_{21}$, we get

$$v_{1}\left(I_{21}\right)=2I_{21}+I_{31},\;v_{2}\left(I_{21}\right)=2I_{31}+I_{32}.$$

This implies that the invariants $I_{21},I_{33},I_{34}$ are sufficient for generating the algebra of differential invariants, together with $v_{1}$ and $v_{2}$. Let us express the differential syzygies relating these generators.

The derivations $v_{1},v_{2}$ satisfy the commutation relation

$$\begin{array}{cc} \left[v_{1},v_{2}\right] & =\left(\frac{\left(I_{33}-I_{42}\right)I_{22}-I_{23}\left(I_{32}-I_{41}\right)}{I_{21}I_{23}-I_{22}^{2}}-3I_{21}\right)v_{1}+\left(\frac{-\left(I_{33}-I_{42}\right)I_{21}+I_{22}\left(I_{32}-I_{41}\right)}{I_{21}I_{23}-I_{22}^{2}}+1\right)v_{2}, \end{array}$$

where $I_{41}=\sigma_{4}\left(v_{1},v_{1},v_{1},v_{1}\right)$ and $I_{42}=\sigma_{4}\left(v_{1},v_{1},v_{1},v_{2}\right)$. The commutation relation determines some of the differential syzygies. When finding the rest of them, we consider only higher-order invariant derivatives of the form $v_{2}^{a_{2}}\circ v_{1}^{a_{1}}$, where $a_{i}$ are non-negative integers.

Invariant derivatives of $I_{21}$ give k−1 differential invariants of order *k* for k≥3 while the invariant derivatives of $I_{33}$ and $I_{34}$ give 2(k−2) differential invariants of order *k* for k≥4. This implies that we get 4 differential invariants of order three, 7 of order four and, in general, 3k−5 of order *k* for k≥4. The Hilbert function is given by $H_{k}=k+1$ for k≥3, implying that there are 2k−6 syzygies for k≥4.

Since the differential invariants are rational, finding the differential syzygies becomes a completely algebraic problem. The first two differential syzygies (for k=4) are found with the help of Maple, and they are given by

$$\begin{array}{c} S_{1}=3J_{4}\;v_{1}\left(v_{1}\left(I_{21}\right)\right)-\left(2J_{1}+2J_{2}+3J_{3}\right)\;v_{2}\left(v_{1}\left(I_{21}\right)\right)+J_{1}\;v_{2}\left(v_{2}\left(I_{21}\right)\right)-v_{2}\left(I_{33}\right)+v_{1}\left(I_{34}\right)\\ -\left(4J_{2}+6J_{4}\right)\;v_{1}\left(I_{21}\right)+\left(4J_{1}+6J_{2}+6J_{3}\right)\;v_{2}\left(I_{21}\right)-J_{2}I_{33}+\left(4+2J_{1}\right)I_{34}+8J_{2}I_{21}=0,\\ S_{2}=-4J_{2}\;v_{1}\left(v_{1}\left(I_{21}\right)\right)+\left(4J_{1}-2\right)\;v_{2}\left(v_{1}\left(I_{21}\right)\right)+v_{2}\left(v_{2}\left(I_{21}\right)\right)-2\;v_{1}\left(I_{33}\right)+I_{34}-4J_{1}I_{33}\\ +\left(8J_{1}-2J_{1}^{2}+6J_{2}\right)\;v_{1}\left(I_{21}\right)+\left(4J_{2}-12J_{1}+4\right)\;v_{2}\left(I_{21}\right)+\left(4J_{1}^{2}+2J_{1}J_{2}+4J_{2}-16J_{1}\right)I_{21}=0. \end{array}$$

Notice that the invariants $J_{i}$ can be expressed in terms of $I_{21},I_{33},I_{34},v_{1}\left(I_{21}\right)$ and $v_{2}\left(I_{21}\right)$.

Since only $S_{1}$ depends on invariant derivatives of $I_{34}$, it is clear that the invariant derivatives of $S_{1}=0$ and $S_{2}=0$ will give 2(k−3) additional independent syzygies involving differential invariants of order *k* for each k≥4. Thus, they generate the desired number of differential syzygies. The commutation relation can be used to ensure that the syzygies are written purely in terms of invariants of the form $v_{2}^{a_{2}}\circ v_{1}^{a_{1}}\left(I\right)$, where *I* is $I_{21}$, $I_{33}$ or $I_{34}$. We get the following theorem.

**Theorem** **7.** *The differential algebra of scalar differential invariants is generated by the invariant derivations $v_{1}$ and $v_{2}$, and the scalar differential invariants $I_{21}$, $I_{33}$ and $I_{34}$. The differential syzygies are generated by $S_{1}=0$ and $S_{2}=0$.*

**Remark** **5.** *In [5] it was noted that there is a relation between the curvature of $\sigma_{2}$ and higher moments. Indeed, when dimV=2 the scalar curvature of $\sigma_{2}$ can be written as*

$$-\frac{1}{2}\frac{\left(I_{21}I_{23}-I_{22}^{2}\right)I_{34}+\left(2I_{31}I_{23}-I_{21}I_{33}\right)I_{33}-I_{32}^{3}}{{\left(I_{21}I_{23}-I_{22}^{2}\right)}^{2}}.$$

## 5. Thermodynamics of Gases

We will now apply the ideas above to the context of gases in thermodynamic equilibrium. Consider the thermodynamic space with variables p,T,e,v,s corresponding to pressure, temperature, internal energy, volume and entropy. The entropy is related to the information gain *I* by the formula dI=−ds. By aligning the one-form $\theta =dI-\lambda_{i}dx^{i}$ with the fundamental thermodynamic relation $-ds+T^{-1}de+pT^{-1}dv=0$, we see that we can get the standard thermodynamics of gases from the measurement of a point (e,v)∈V, and the principle of minimal information gain. The relationship between p,T,e,v and $x^{1},x^{2},\lambda_{1},\lambda_{2}$ is

$$x_{1}=e,\;x_{2}=v,\;\lambda_{1}=-T^{-1},\;\lambda_{2}=-pT^{-1}.$$

We will suppress the information gain, or entropy, from the picture, and consider a system in thermodynamic equilibrium as a Lagrangian submanifold of $V\times V^{*}$ on which the symplectic form

$$\omega =d\theta =\frac{1}{T^{2}}\left(de\wedge dT+pdv\wedge dT+Tdp\wedge dv\right)$$

vanishes. We will assume T≠0 throughout.

Similarly as above, we let the Lagrangian submanifold $L\subset V\times V^{*}$ be given by two functions e(T,p),v(T,p). Restricting ω to such a submanifold gives

$${\omega |}_{L}=\frac{1}{T^{2}}\left(e_{p}+pv_{p}+Tv_{T}\right)dp\wedge dT,$$

implying that *L* is Lagrangian if and only if

$$F=e_{p}+Tv_{T}+pv_{p}=0.$$

The differential equation F=0 determines the submanifold $E_{1}$ in $J^{1}\left(V^{*}\times V,2\right)$.

### 5.1. The Group Action

In the (nonlinear) coordinates T,p,e,v, the Lie algebra corresponding to the Aff(V) action on $V\times V^{*}$ is spanned by the six vector fields

$$\begin{array}{c} \partial_{e},\;\partial_{v},\;\partial_{p}-v\partial_{e},\;e\partial_{e}+T\partial_{T}+p\partial_{p},\;v\partial_{v}-p\partial_{p},\;e\partial_{v}+Tp\partial_{T}+p^{2}\partial_{p}. \end{array}$$

Orbits in general position in $E_{1}$ are six-dimensional. The subset on which the orbit dimension decreases is given by $T(e_{p}v_{T}-e_{T}v_{p})=0$. Positive definiteness of $\sigma_{2}$ implies positivity of the left-hand side of this equation (see the end of Section 5.2).

One can ask if it is appropriate to consider arbitrary affine transformation on points (e,v). We will consider the action by two different subgroups of Aff(V), in addition to the full group of affine transformations, and write down the differential invariants for each of these three group actions. The choices of the two subgroups are based on recent results concerning the thermodynamics of fluids on two different manifolds.

In [17], the symmetries of compressible viscid fluids were found. Some of the symmetries are purely geometrical, such as translations, rotations and Galilean transformations. In addition there are some symmetries that act on thermodynamic variables. In particular there is a three-dimensional Lie algebra that is spanned by

$$e\partial_{e}+T\partial_{T}+p\partial_{p},\;v\partial_{v}-p\partial_{p},\;v\partial_{e}-\partial_{p}.$$

It corresponds to the group action given by (e,v)↦(Ae+Cv,Bv) for A,B∈R\{0}, C∈R.

In [18], the symmetries of compressible viscid fluids on a spherical layer were found. In this case, the thermodynamic part of the symmetry Lie algebra is a Lie subalgebra of the one above. It is spanned by

$$v\partial_{e}-\partial_{p},\;v\partial_{v}+2e\partial_{e}+2T\partial_{T}+p\partial_{p}.$$

It corresponds to the group action given by $\left(e,v\right)\mapsto \left(A^{2}e+Bv,Av\right)$ for A∈R\{0}, B∈R.

**Remark** **6.** To be precise, in both [17,18] there is also a vector field $\partial_{s}$ corresponding to translation in entropy. These transformations are not contained in Aff(V), and since we consider here only differential invariants of Lagrangian submanifolds of $V\times V^{*}$, they will not affect our description of differential invariants. However, we swiftly note that if we extend the Lie algebras above with this vector field, the differential invariant of order zero that corresponds to entropy will not be invariant any more.

### 5.2. Differential Invariants with Respect to Aff(V)

The algebra of differential invariants with respect to Aff(V) was in principle completely described in Section 4. We found both generators and syzygies for the case dimV=2. In the new coordinates, the tensors $\alpha_{1}$, $\sigma_{2}$ and $\sigma_{3}$ are given by

$$\begin{array}{cc} \alpha_{1} & =-\frac{1}{T}\left(\left(pv_{T}+e_{T}\right)dT-Tv_{T}dp\right),\\ \sigma_{2} & =\frac{1}{T^{2}}\left(\left(pv_{T}+e_{T}\right)dT^{2}-2Tv_{T}dTdp-Tv_{p}dp^{2}\right),\\ \sigma_{3} & =\frac{1}{T^{3}}(\left(2\left(pv_{T}+e_{T}\right)+T\left(pv_{TT}+e_{TT}\right)\right)dT^{3}-3T\left(Tv_{TT}+2v_{T}\right)dT^{2}dp\\ & \;-3T\left(Tv_{Tp}+v_{p}\right)dTdp^{2}-T^{2}v_{pp}dp^{3}). \end{array}$$

In this section we will use a slightly different set of generators for the algebra of differential invariants. With the help of the DifferentialGeometry and JetCalculus packages and the pdsolve procedure in Maple we find a generating set of invariants whose expressions are simpler in the current choice of coordinates. It is possible to find the second-order differential invariants by solving the system of linear partial differential equations given by $X_{i}^{\left(1\right)}\left(f\left(T,p,e,v,e_{T},v_{T},v_{p}\right)\right){|}_{F=0}=0$ for a basis $X_{1},...,X_{6}$ of the Lie algebra of vector fields corresponding to the group action.

First of all, we have the following invariant derivations:

$$\nabla_{1}=-TD_{T},\;\nabla_{2}=\frac{Tv_{T}D_{T}+\left(e_{T}+pv_{T}\right)D_{p}}{(e_{T}v_{TT}-e_{TT}v_{T})T}$$

And we remember from the previous section that the Hilbert function for the field of rational differential invariants is given by $H_{1}=0,H_{2}=1$ and $H_{k}=k+1$ for k≥3.

The verification of the theorems in this section consists of two parts. Firstly, one must check that the functions and derivations given are invariant, something we recommend doing with the help of Maple. Secondly, one must verify that the differential invariants and invariant derivations allow us to generate a sufficient amount of independent differential invariants (compare with $H_{k}$). Due to the simple form of the differential invariants and invariant derivations, this is straightforward.

**Theorem** **8.** *The differential invariants*

$$\begin{array}{c} I_{2}=pv_{T}+e_{T},\;I_{31}=-\left(pv_{TT}+e_{TT}\right)T,\;I_{32}=\frac{{\left(e_{T}v_{TT}-e_{TT}v_{T}\right)}^{2}T^{3}}{e_{p}v_{T}-e_{T}v_{p}},\\ I_{33}=\frac{\left(2Tv_{T}I_{2}v_{TT}+I_{2}^{2}v_{Tp}+v_{T}^{2}\left(I_{2}+I_{31}\right)\right)T}{\left(e_{p}v_{T}-e_{T}v_{p}\right)},\\ I_{34}=\frac{T^{2}\left(3T^{2}v_{T}^{2}I_{2}v_{TT}+3Tv_{T}I_{2}^{2}v_{Tp}+I_{2}^{3}v_{pp}+Tv_{T}^{3}I_{31}+4Tv_{T}^{3}I_{2}+3v_{T}v_{p}I_{2}^{2}\right)\left(e_{T}v_{TT}-e_{TT}v_{T}\right)}{{\left(e_{p}v_{T}-e_{T}v_{p}\right)}^{2}} \end{array}$$

*constitute a transcendence basis for the field of third-order differential invariants.*

Here we have recycled the notation $I_{3i}$ that we used in Section 4.3. These are not exactly the generators we used above, but $I_{2}=\sigma_{2}\left(v_{1},v_{1}\right)$. We notice that this second-order differential invariant is what is known in thermodynamics as the heat capacity (at constant pressure). Thus the concept of heat capacity is given to us automatically if we consider the action of the affine group on $V\times V^{*}$ (a subgroup of the affine group will lead to more scalar differential invariants).

**Example** **1.** *Requiring $I_{2}$ to be constant singles out a special set of Lagrangian submanifolds. They are solutions to the system*

$$F=e_{p}+Tv_{T}+pv_{p}=0,\;I_{2}=pv_{T}+e_{T}=C$$

*for C∈R, and they are given by*

$$e=f_{1}\left(p\right)T-f_{2}^{\prime }\left(p\right)p^{2},\;v=\frac{(C-f_{1}\left(p\right))T}{p}+f_{2}^{\prime }\left(p\right)p+f_{2}\left(p\right).$$

By using the invariant derivations $\nabla_{1}$ and $\nabla_{2}$ we can generate the algebra of differential invariants. Notice that $I_{31}=\nabla_{1}\left(I_{2}\right)$.

**Theorem** **9.** *The differential algebra of scalar differential invariants is generated by the invariant derivations $\nabla_{1}$ and $\nabla_{2}$, together with the differential invariants $I_{2},I_{32},I_{33},I_{34}$.*

Notice also that the tensor $\sigma_{2}$ takes diagonal form when written in terms of the frame $\nabla_{1},\nabla_{2}$:

$$\sigma_{2}\left(\nabla_{1},\nabla_{1}\right)=I_{2},\;\sigma_{2}\left(\nabla_{1},\nabla_{2}\right)=0,\;\sigma_{2}\left(\nabla_{2},\nabla_{2}\right)=I_{2}/I_{32}$$

Positive definiteness of $\sigma_{2}$ implies $I_{2}>0$ and $I_{32}>0$ (the latter may be replaced by $(e_{p}v_{T}-e_{T}v_{p})T>0$).

### 5.3. Differential Invariants with Respect to a Three-Dimensional Subgroup

Now, let us consider the three-dimensional group from [17] which acts by (e,v)↦(Ae+Cv,Bv) for A,B∈R\{0}, C∈R.

The Hilbert function is in this case given by $H_{1}=1$ and $H_{k}=k+1$ for k≥2. Thus, we get 4 invariants of order two.

**Theorem** **10.** *The differential invariants*

$$\begin{array}{c} \frac{e+pv}{T},\;\frac{Tv_{T}}{v},\;e_{T}+pv_{T},\;e_{T}-e_{p}\frac{v_{T}}{v_{p}} \end{array}$$

*generate the field of second-order differential invariants.*

We notice that the last two invariants are the heat capacity at constant pressure and at constant volume, respectively. The derivations $TD_{T}$ and $\frac{T}{v}D_{p}$ are invariant, and by using them we can generate the algebra of differential invariants.

**Theorem** **11.** *The differential algebra of differential invariants is generated by the following two differential invariants of order 1 and two invariant derivations:*

$$J_{1}=\frac{e+pv}{T},\;J_{2}=\frac{Tv_{p}}{v^{2}},\;{\tilde{\nabla }}_{1}=TD_{T},\;{\tilde{\nabla }}_{2}=\frac{T}{v}D_{p}$$

*The generators are related by the differential syzygy*

$${\tilde{\nabla }}_{1}\left(J_{2}\right)+{\tilde{\nabla }}_{2}\left({\tilde{\nabla }}_{2}\left(J_{1}\right)\right)-J_{2}{\tilde{\nabla }}_{2}\left(J_{1}\right)=0.$$

The invariant $J_{2}$ can be written in terms of the invariants in Theorem 10. We use it here because it gives a simple differential syzygy.

**Example** **2.** *Let us find the Lagrangian submanifolds for which $J_{1}$ is constant. By solving the system*

$$F=e_{p}+Tv_{T}+pv_{p}=0,\;J_{1}=\frac{e+pv}{T}=C,$$

*for C∈R, we get*

e=(C−f(p))T,pv=f(p)T.

### 5.4. Differential Invariants with Respect to a Two-Dimensional Subgroup

Now, let us consider the two-dimensional Lie group from [18] which acts by $\left(e,v\right)\mapsto \left(A^{2}e+Bv,Av\right)$ for A∈R\{0}, B∈R.

The Hilbert function is in this case given by $H_{k}=k+1$ for k≥1. Thus, we get 5 invariants of order two.

**Theorem** **12.** *The differential invariants*

$$\begin{array}{c} \frac{pv+e}{T},\;\frac{v^{2}}{T},\;vv_{T},\;v_{p},\;pv_{T}+e_{T} \end{array}$$

*generate the field of second-order differential invariants.*

The derivations $TD_{T}$ and $vD_{p}$ are invariant, and by using them we can generate the algebra of differential invariants. We reuse the notation from the previous subsection.

**Theorem** **13.** *The differential algebra of differential invariants is generated by the following two differential invariants of order 1 and two invariant derivations:*

$$J_{1}=\frac{pv+e}{T},\;J_{2}=\frac{v^{2}}{T},\;{\tilde{\nabla }}_{1}=TD_{T},\;{\tilde{\nabla }}_{2}=vD_{p}$$

*The generators are related by the differential syzygy*

$$2{\tilde{\nabla }}_{2}\left(J_{1}\right)+{\tilde{\nabla }}_{1}\left(J_{2}\right)-J_{2}=0.$$

### 5.5. The Significance of the Differential Invariants

We end the paper with a discussion on the significance of the differential invariants. In order to simplify the discussion, we will focus mainly on the group action of the two-dimensional Lie group considered in the previous subsection, but the ideas are general. We denote the Lie group by *G*.

A differential invariant *I* is a function on $E_{k}\subset J^{k}\left(V\times V^{*},2\right)$ that is constant on *G*-orbits. It can be restricted to a Lagrangian submanifold *L*, resulting in a function ${I|}_{L}$ on *L*. For example, if *L* is given by a particular pair of functions e(T,p) and v(T,p), then the differential invariant $e_{T}+pv_{T}$ restricts to a function $e_{T}\left(T,p\right)+pv_{T}\left(T,p\right)$. A transformation from the Lie group under consideration, such as $\left(\tilde{T},\tilde{p},\tilde{e},\tilde{v}\right)=\left(T,p-A,e+Av,v\right)$, transforms the functions e(T,p) and v(T,p) into two new functions $\tilde{e}\left(\tilde{T},\tilde{p}\right)=e\left(T,p-A\right)-Av\left(T,p-A\right)$ and $\tilde{v}\left(\tilde{T},\tilde{p}\right)=v\left(T,p-A\right)$, but the differential invariant remains the same:

$${\tilde{e}}_{T}\left(\tilde{T},\tilde{p}\right)+\tilde{p}{\tilde{v}}_{T}\left(T,p\right)=e_{T}\left(T,p-A\right)+\left(p-A\right)v_{T}\left(T,p-A\right)$$

This function gives the same value at the point (T,p) in *L* as at the point $\left(\tilde{T},\tilde{p}\right)$ in the transformed Lagrangian manifold $\tilde{L}$. Thus, if we treat Lagrangian manifolds that are related by a transformation in *G* as equivalent, the differential invariants play an important role, as they are the scalar functions determined by e(T,p) and v(T,p) and their derivatives that only depend on the equivalence class of *L*.

The differential invariants can also be used to distinguish non-equivalent Lagrangian manifolds and classify them. Consider the differential invariants

$$J_{1}=\frac{pv+e}{T},\;J_{2}=\frac{v^{2}}{T},\;K_{1}=vv_{T},\;K_{2}=v_{p},\;K_{3}=pv_{T}+e_{T}.$$

If we restrict these invariants to a Lagrangian submanifold *L*, we get five functions that depend on *T* and *p*. We consider the subset of $R^{5}$ parametrized by $\left(J_{1}{|}_{L}\left(T,p\right),J_{2}{|}_{L}\left(T,p\right),K_{1}{|}_{L}\left(T,p\right),K_{2}{|}_{L}\left(T,p\right),K_{3}{|}_{L}\left(T,p\right)\right)$. For generic Lagrangian manifolds this is a two-dimensional surface, and we call it the signature surface of *L*. Clearly, two equivalent Lagrangian manifolds give the same signature surface.

We will now generate the algebra of differential invariants in a different way than we did above. Let ${\hat{\partial }}_{1},{\hat{\partial }}_{2}$ denote the Tresse derivatives with respect to the pair $J_{1},J_{2}$. They are derivations of the form $aD_{T}+bD_{p}$, where *a* and *b* are functions on $E_{1}$, that are uniquely determined by the condition ${\hat{\partial }}_{i}\left(J_{j}\right)=\delta_{ij}$. Here it is important that

$$\hat{d}J_{1}\wedge \hat{d}J_{2}=\frac{v}{T^{2}}\left(J_{2}-2J_{1}K_{2}-3K_{1}+2K_{2}K_{3}+2K_{1}^{2}/J_{2}\right)dT\wedge dp\neq 0,$$

where $\hat{d}$ is the horizontal differential. This condition determines an Zariski-open subset in $E_{1}$, and we consider only Lagrangian manifolds whose 1-jets lie in this open subset. Another way to say it is that we consider only Lagrangian manifolds *L* such that $d\left(J_{1}{|}_{L}\right)\wedge d\left(J_{2}{|}_{L}\right)\neq 0$ for every point in *L*. Note that this condition guarantees that the signature surfaces are two-dimensional.

It is not difficult to check that we can generate all differential invariants by using these five differential invariants and the Tresse derivatives. Then, since all differential invariants can be generated from $J_{1},J_{2},K_{1},K_{2},K_{3}$ and the Tresse derivatives, it becomes clear that the signature manifold of *L* contains all information about its invariants. Since the field of rational differential invariants separates orbits in general position in $E_{k}$ for every *k* [14], distinguishing non-equivalent Lagrangian manifolds (with jets in general position) comes down to comparing their signature surfaces.

Now we will find conditions that the signature surfaces must satisfy. By computing ${\hat{\partial }}_{i}\left(K_{j}\right)$, we get six differential invariants of order three. Since $H_{3}=4$, there are at least two differential syzygies. In fact, there are exactly two and they are given by

$$\begin{array}{cc} 0=( & \left(-2J_{2}K_{2}\left(J_{1}-K_{3}\right)+\left(J_{2}-K_{1}\right)\left(J_{2}-2K_{1}\right)\right){\hat{\partial }}_{1}\left(K_{1}\right)+J_{2}\left(J_{2}-2K_{1}\right)\left(J_{1}-K_{3}\right){\hat{\partial }}_{1}\left(K_{2}\right)\\ & +J_{2}{\left(J_{2}-2K_{1}\right)}^{2}{\hat{\partial }}_{2}\left(K_{2}\right)+2J_{2}K_{2}\left(J_{2}-K_{1}\right){\hat{\partial }}_{1}\left(K_{3}\right)+4J_{2}^{2}K_{2}^{2}{\hat{\partial }}_{2}\left(K_{3}\right)-J_{2}K_{1}K_{2}),\\ 0=( & \left(2J_{2}^{2}K_{2}\left(J_{1}-K_{3}\right)-J_{2}\left(J_{2}-K_{1}\right)\left(J_{2}-2K_{1}\right)\right){\hat{\partial }}_{2}\left(K_{1}\right)+J_{2}^{2}{\left(J_{1}-K_{3}\right)}^{2}{\hat{\partial }}_{1}\left(K_{2}\right)\\ \; & +J_{2}^{2}\left(J_{2}-2K_{1}\right)\left(J_{1}-K_{3}\right){\hat{\partial }}_{2}\left(K_{2}\right)+J_{2}{\left(J_{2}-K_{1}\right)}^{2}{\hat{\partial }}_{1}\left(K_{3}\right)+2J_{2}^{2}K_{2}\left(J_{2}-K_{1}\right){\hat{\partial }}_{2}\left(K_{3}\right)\\ & -K_{1}\left(K_{1}\left(J_{2}-K_{1}\right)+J_{2}K_{2}\left(J_{1}-K_{3}\right)\right)). \end{array}$$

All higher-order syzygies are differential consequences of these. The Tresse derivatives play the role of partial derivatives with respect to $J_{1}$ and $J_{2}$ (their restriction to a Lagrangian submanifold *L* are partial derivatives with respect to $J_{1}{|}_{L}$ and $J_{2}{|}_{L}$), and these two differental syzygies are partial differential equations that the signature manifolds must satisfy. They are often called the quotient or factor equations. Their solutions can be interpreted as equivalence classes of Lagrangian submanifolds.
